# Accumulation of synovial fluid CD19^+^CD24^hi^CD27^+^ B cells was associated with bone destruction in rheumatoid arthritis

**DOI:** 10.1038/s41598-020-71362-7

**Published:** 2020-09-01

**Authors:** Xiaofeng Guo, Tingting Xu, Jing Zheng, Xiangjun Cui, Ming Li, Kai Wang, Min Su, Huifang Zhang, Ke Zheng, Chongling Sun, Shulin Song, Hongjiang Liu

**Affiliations:** 1 Department of Rheumatology and Immunology, The People’s Hospital of China Three Gorges University/The First People’s Hospital of Yichang, No. 4, Hudi Street, Xiling District, Yichang, 443000 Hubei Province China; 2 Department of Hematology, The People’s Hospital of China Three Gorges University/The First People’s Hospital of Yichang, Yichang, 443000 Hubei Province China

**Keywords:** Autoimmunity, Rheumatoid arthritis

## Abstract

Regulatory CD19^+^CD24^hi^CD27^+^ B cells were proved to be numerically decreased and functionally impaired in the peripheral blood (PB) from rheumatoid arthritis (RA), with the potential of converting into osteoclast-priming cells. However, the distribution and function of CD19^+^CD24^hi^CD27^+^ B cells in RA synovial fluid (SF) were unclear. In this study, we investigated whether RA SF CD19^+^CD24^hi^CD27^+^ B cells were increased and associated with bone destruction. We found that the proportion of RA SF CD19^+^CD24^hi^CD27^+^ B cells was increased significantly, and was positively correlated with swollen joint counts, tender joint counts and disease activity. CXCL12, CXCL13, CCL19 contributed to the recruitment of CD19^+^CD24^hi^CD27^+^ B cells in RA SF. Notably, CD19^+^CD24^hi^CD27^+^ B cells in the SF from RA expressed significantly more RANKL compared to OA and that in the PB from RA. Critically, RA CD19^+^CD24^hi^CD27^+^ B cells promoted osteoclast (OC) differentiation in vitro, and the number of OCs was higher in cultures with RA SF CD19^+^CD24^hi^CD27^+^ B cells than in those derived from RA PB. Collectively, these findings revealed the accumulation of CD19^+^CD24^hi^CD27^+^ B cells in SF and their likely contribution to joint destruction in RA. Modulating the status of CD19^+^CD24^hi^CD27^+^ B cells might provide novel therapeutic strategies for RA.

## Introduction

Rheumatoid arthritis (RA) is a common chronic autoimmune disease characterized by synovitis in multiple joints and progressive bone destruction^1^. Mounting evidence indicates that the imbalance between bone loss and bone formation attributes to bone damage in RA^2^. Bone-resorbing osteoclasts (OCs) are the cells responsible for bone erosion in RA patients^3^. Receptor activator of NF-κB ligand (RANKL) and its receptor, RANK, are key positive extracellular regulators of osteoclast formation and activation^4,5^.

Early studies of RANKL production in RA indicated that synovial fibroblasts and activated T cells produce excess RANKL and may contribute to osteoclastic bone resorption^6–8^. However, recent increasing researches have demonstrated that B cells play an important role in facilitating bone erosion in RA by both promoting OC differentiation and by suppressing osteoblast (OB) development^9–13^. Scientists found that synovial B cells are a major source of RANKL and Fc-receptor like 4 (FcRL4) positive B cells are defined a pro-inflammatory, RANKL-producing B cells in RA^9,10^. Thereafter, switched-memory B cells have been reported in RA peripheral blood and synovial tissue, where they are thought to express high level of RANKL and promoted osteoclastogenesis^11,12^. Moreover, B cells have been proved to inhibit bone formation in RA by secreting multiple OB inhibitors^13^, furtherly indicating the tight relationship between B lymphocytes and bone homeostasis.

As reported, CD19^+^CD24^hi^CD27^+^ B cells were identified as IL-10 producing B cell subsets in human, which also termed as regulatory B10 cells^14^. Several studies have shown that these B10 cells were numerically decreased and functionally impaired in the peripheral blood (PB) of RA and some other autoimmune disease^15–17^. Our previous study has shown that PB CD19^+^CD24^hi^CD27^+^ B cells have the potential of converting into RANKL-producing cells in RA patients^18^. These findings highlight the important role of B cells in modulating bone homeostasis during inflammatory arthritis. Although recent studies demonstrated that B cells produce RANKL in RA, there is a lack of consensus on which specific B-cell subsets exacerbate bone destruction in the local joints. However, characteristics of CD19^+^CD24^hi^CD27^+^ B cells in RA patient synovial fluids remain unclear.

The underlying mechanisms of the numerical and functional changes in PB CD19^+^CD24^hi^CD27^+^ B cells in RA patients are unknown. One possible hypothesis is that under pathological conditions of RA, PB CD19^+^CD24^hi^CD27^+^ B cells were recruited to SF and might convert into pathogenic cells. During synovial inflammation, leukocyte trafficking to synovial fluids is a key pathogenic process in RA, in particular T cells and B cells, which is mediated by chemokines and chemokine receptors^19–21^. B cell-related chemokines, CXCL10, CXCL12, CXCL13, CCL19, CCL20 and CCL21, might contribute to the recruitment and maintenance of B cells in arthritic joints^22–24^, which needs to be identified in synovial fluid from RA patients.

This study was performed to characterize the distribution pattern of CD19^+^CD24^hi^CD27^+^ B cells in RA SF, and to reveal their bone-destructive capacities.

## Results

### SF CD19^+^CD24^hi^CD27^+^ B cells were increased in RA patients and were inversely correlated with that in PB

**Figure 1:**
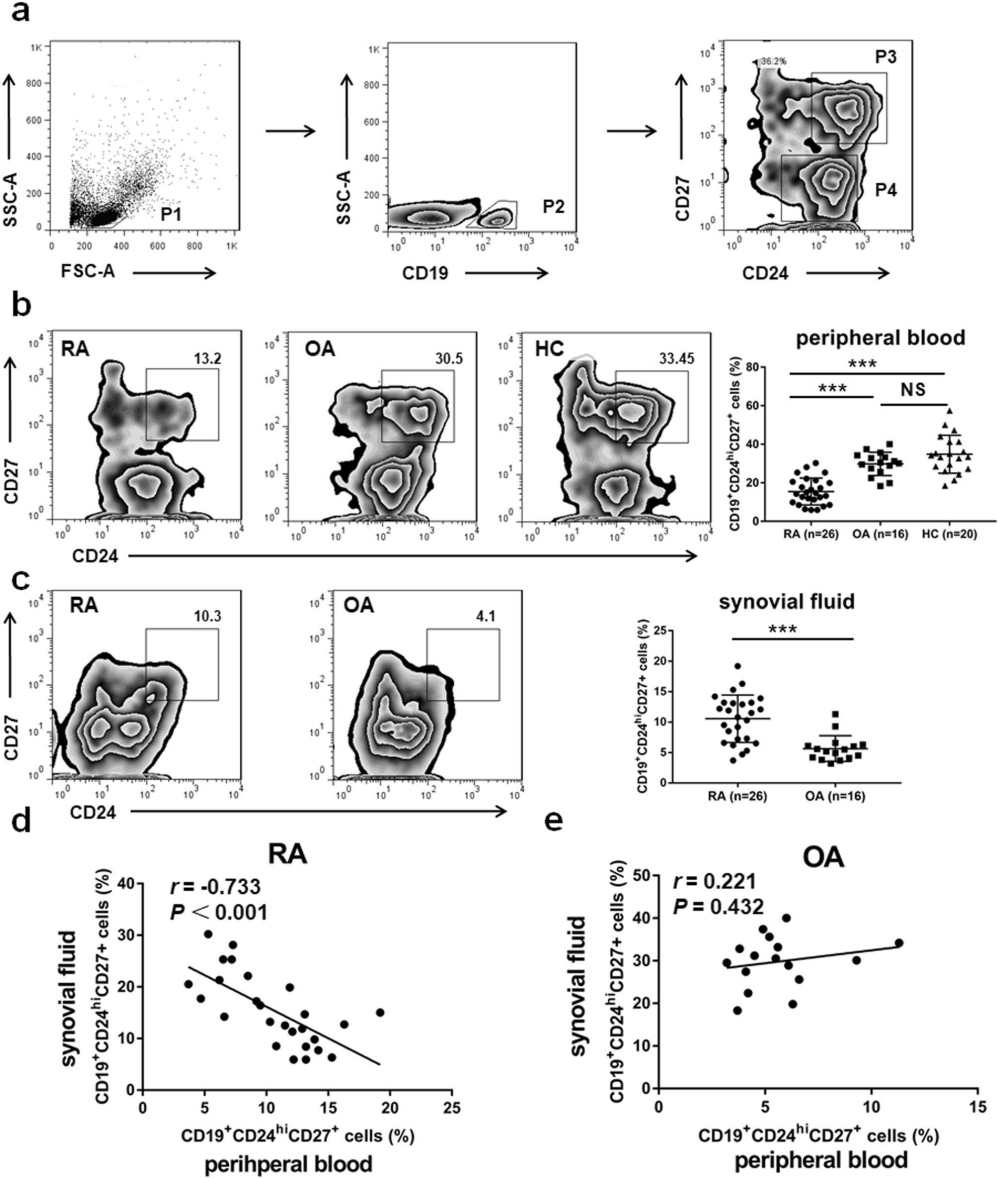
Increased SF CD19^+^CD24^hi^CD27^+^ B cells were inversely correlated with PB CD19^+^CD24^hi^CD27^+^ B cells in patients with RA. Representative flow cytometry charts depicting the gating strategy for CD19^+^CD24^hi^CD27^+^ B cells (P3) in healthy PB (**a**). The percentage of PB CD19^+^CD24^hi^CD27^+^ B cells was analyzed in 26 patients with RA, 16 patients with OA and 20 healthy controls (HC) (**b**). The proportion of SF CD19^+^CD24^hi^CD27^+^ B cells was assessed by flow cytometric analysis in 26 RA and 16 OA patients (**c**). The correlations between the percentage of SF CD19^+^CD24^hi^CD27^+^ B cells and paired PB CD19^+^CD24^hi^CD27^+^ B cells of RA patients (n = 26) (**d**) and OA patients (n = 16) (**e**) were evaluated respectively. ***p < 0.001; NS, not significant.

To evaluate the distribution of CD19^+^CD24^hi^CD27^+^ B cells in SF of RA patient, we collected SF and paired PB of patients with RA and OA. We first measured by flow cytometric analysis the proportion of CD19^+^CD24^hi^CD27^+^ B cells in the PB from RA and OA patients and HC. A representative experiment of the flow-cytometric gating strategy of CD19^+^CD24^hi^CD27^+^ B cell subsets in PB mononuclear cells (PBMCs) from a healthy control was shown (Fig. 1a). There was no significant change of the proportion of PB CD19^+^CD24^hi^CD27^+^ B cells between HC and OA patients. However, the percentage of PB CD19^+^CD24^hi^CD27^+^ B cells was significantly decreased in RA patients as compared with OA and healthy individuals (Fig. 1b).

Then we evaluated the proportion of CD19^+^CD24^hi^CD27^+^ B cells in SF from patients with RA and OA. Indeed, we observed a markedly higher percentage of CD19^+^CD24^hi^CD27^+^ B cells in the SFMCs of patients with RA as compared to patients with OA (Fig. 1c). Then, we asked whether the expansion of SF CD19^+^CD24^hi^CD27^+^ B cells in RA patient might lead to the reduction of PB CD19^+^CD24^hi^CD27^+^ B cells in RA patients. To further demonstrate the hypothesis, we have also analyzed the proportion of CD19^+^CD24^hi^CD27^+^ B cells in SF and paired PB of RA and OA patients. We found that the proportion of CD19^+^CD24^hi^CD27^+^ B cells in RA SF was negatively correlated with the frequencies of CD19^+^CD24^hi^CD27^+^ B cells in RA PB (Fig. 1d). There was no correlation between CD19^+^CD24^hi^CD27^+^ B cells in SF and PB of OA patients (Fig. 1e). These results suggested that PB CD19^+^CD24^hi^CD27^+^ B cells may migrate to SF in RA patients. In summary, the accumulation of CD19^+^CD24^hi^CD27^+^ B cells in SF of RA patients is probably due to the migration of PB CD19^+^CD24^hi^CD27^+^ B cells.

### CXCL12, CXCL13 and CCL19 contributed to the recruitment of CD19^+^CD24^hi^CD27^+^ B cells in the SF of RA patients

**Figure. 2:**
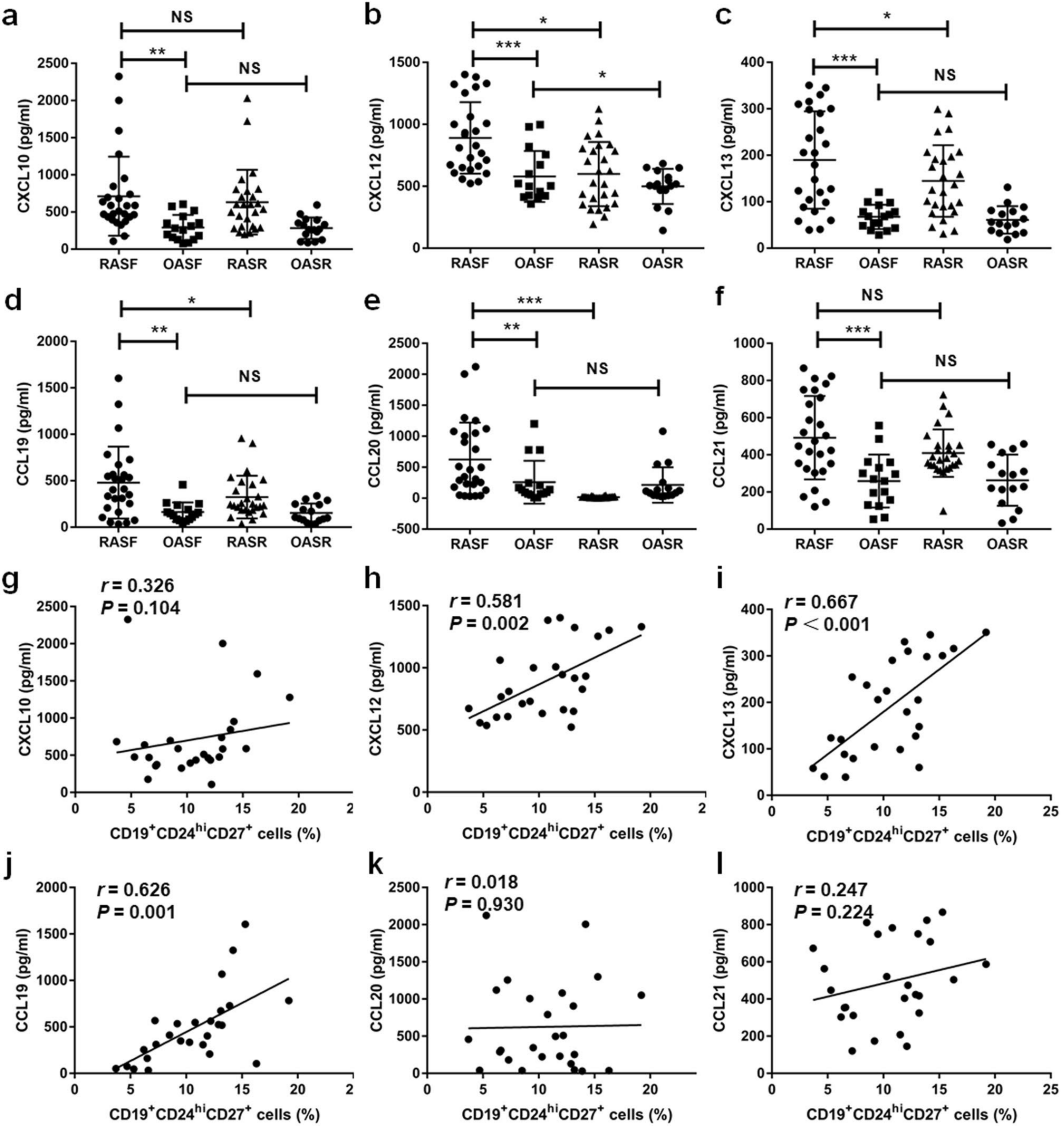
Elevated levels of SF CXCL12, CXCL13, CCL19 were positively correlated with SF CD19^+^CD24^hi^CD27^+^ B cells in RA patients. Levels of CXCL10 (**a**), CXCL12 (**b**), CXCL13 (**c**), CCL19 (**d**), CCL20 (**e**) and CCL21 (**f**) in RA and OA synovial fluid and RA and OA serum were measured by ELISA. The correlations between SF CXCL10 (**g**), CXCL12 (**h**), CXCL13 (**i**), CCL19 (**j**), CCL20 (**k**) and CCL21 (**i**) and the percentage of SF CD19^+^CD24^hi^CD27^+^ B cells were evaluated respectively in RA patients.

While it is likely that multiple factors contribute to the accumulation of CD19^+^CD24^hi^CD27^+^ B cells in RA SF, we were especially interested in whether chemokines may play an important role in the process. The limited studies that do exist suggest that chemokines can directly promote the recruitment of B cells to inflammatory foci in chronic arthritis^24^. Therefore, we hypothesized that the increase of CD19^+^CD24^hi^CD27^+^ B cells in SF may be due to the migration of CD19^+^CD24^hi^CD27^+^ B cells from PB to SF, which can partly explain the decrease in the number of CD19^+^CD24^hi^CD27^+^ B cells in PB of RA patients. B cell-related chemokines were measured by ELISA. Indeed, the levels of CXCL10, CXCL12, CXCL13, CCL19, CCL20 and CCL21 in SF of patients with RA were significantly higher than those in patients with OA (Fig. 2a–f). Further, we found a significant increase in CXCL12, CXCL13, CCL19, and CCL20 in RA SF as compared to paired RA serum (Fig. 2b–e). Finally, we found that levels of RA SF CXCL12, CXCL13 and CCL19 were positively correlated with the proportion of CD19^+^CD24^hi^CD27^+^ B cells in RA SF (Fig. 2h–j). There was no significant correlation between levels of CXCL10, CCL20, and CCL21 and the proportion of CD19^+^CD24^hi^CD27^+^ B cells in SF from RA patients (Fig. 2g, k, l). Levels of these chemokines in OA patients were not significantly different between SF and serum, except for CXCL12 which was higher in SF than that in serum (Fig. 2b–e). In summary, the levels of CXCL12, CXCL13 and CCL19 in synovial fluid of RA patients were significantly higher as compared to RA sera and OA SF, and were positively associated with the proportions of CD19^+^CD24^hi^CD27^+^ B cells in RA SF, suggesting that CXCL12, CXCL13, and CCL19 promote the increase of SF CD19^+^CD24^hi^CD27^+^ B cells in RA patients.

### Correlation analysis of SF CD19^+^CD24^hi^CD27^+^ B cells with RA patient clinical features

**Figure 3:**
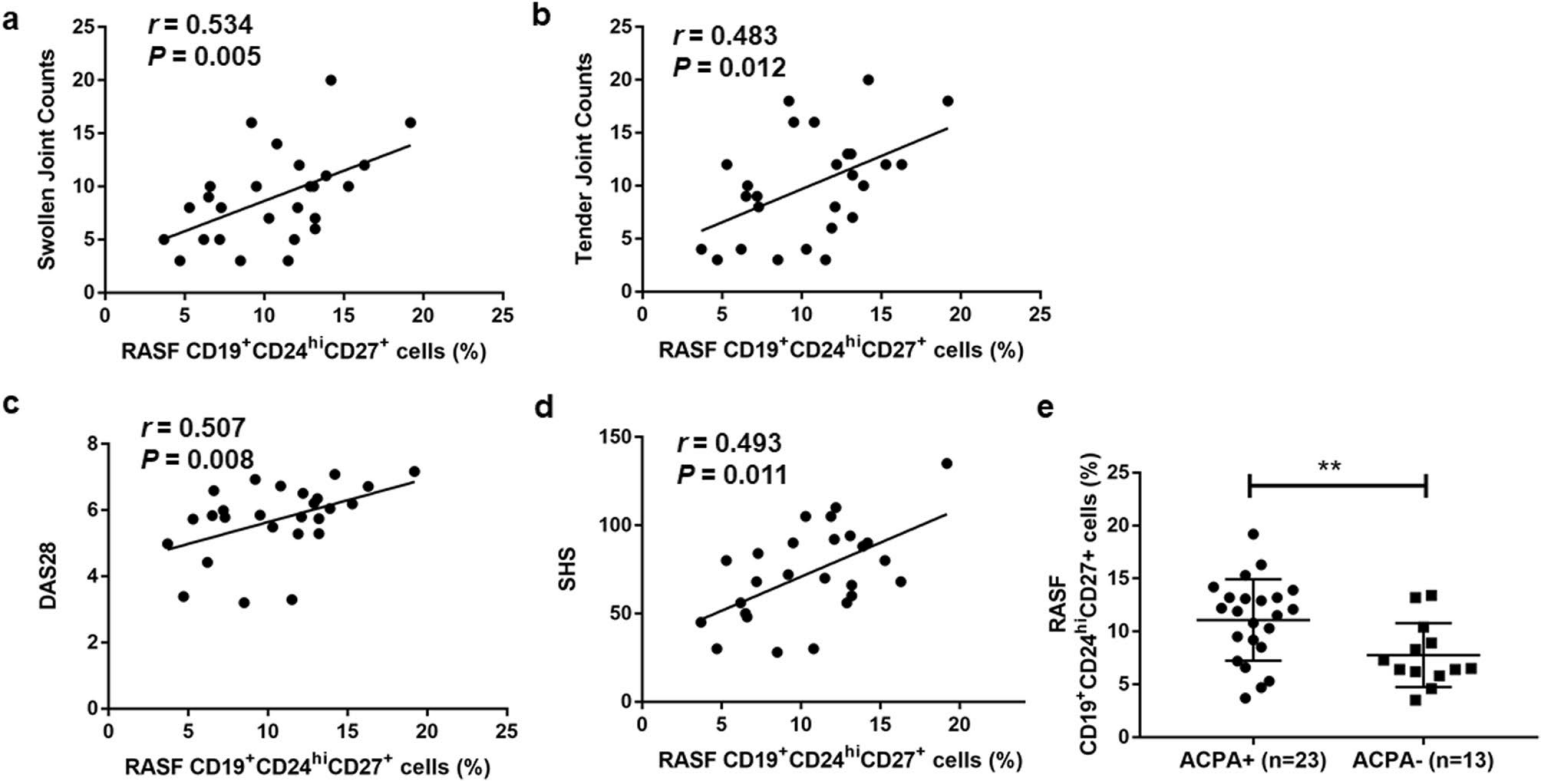
Associations between the proportion of RASF CD19^+^CD^hi^CD27^+^ B cells and RA clinical features. The percentage of RASF CD19^+^CD24^hi^CD27^+^ B cells was positively correlated with swollen joint counts (**a**), tender joint counts (**b**), DAS28 (disease activity score in 28 joints) (**c**) and SHS (**d**). RA patients were divided into ACPA+ group and ACPA− group, then the frequencies of SF CD19^+^CD24^hi^CD27^+^ B cells were compared (**e**). **p < 0.01.

Next, we analyzed the associations between the percentage of CD19^+^CD24^hi^CD27^+^ B cells in SF and clinical and laboratory parameters of RA patients. Clinical characteristics of twenty-six patients with RA were showed in Table 1. The percentages of SF CD19^+^CD24^hi^CD27^+^ B cells were positively correlated with swollen joint counts, tender joint counts, the disease activity score in 28 joints (DAS28), and the radiographic score of joint damage SHS (Fig. 3a–d). No significant correlation was observed between these cells and erythrocyte sedimentation rate (ESR), C-reactive protein (CRP), anti-citrullinated peptide antibody (ACPA), rheumatoid factor (RF) and RA patient age, disease duration (data not shown). To further study the relationship between SF CD19^+^CD24^hi^CD27^+^ B cells and ACPA, we collected synovial fluid from 10 additional ACPA− RA patients (clinical characteristics not shown). ACPA+ RA patients demonstrated higher frequencies of SF CD19^+^CD24^hi^CD27^+^ B cells than ACPA− patients (Fig. 3e). In summary, SF CD19^+^CD24^hi^CD27^+^ B cells were associated with inflammation of the joints and radiographic severity of RA. Our results indicated that SF CD19^+^CD24^hi^CD27^+^ B cells might aggravate the inflamed joint bone destruction in RA patients.

**Table 1:** Clinical characteristics of RA patients.

Characteristics	RA (n = 26)
Age, mean (range), years	55.9 (32–79)
Sex, no. female/male	22/4
Duration, mean (range), years	9.4 (1–30)
Tender joint count, mean (range) of 28 joints	10.03 (3–20)
Swollen joint count, mean (range) of 28 joints	8.96 (3–20)
ESR, mean (range), mm/h	53.69(7–112)
CRP, mean (range), mg/l	36.3 (1.57–119)
DAS28, mean (range)	5.31 (3.20–7.17)
RF, no. positive/no. negative/no. nd	21/5/0
ACPA, no. positive/no. negative/no. nd	23/3/0
SHS, mean (range)	73.08 (28–135)

ESR, erythrocyte sedimentation rate; CRP, C-reactive protein; RF, rheumatoid factor; ACPA, anti-cyclic citrullinated peptide antibody; SHS, modified Sharp/van der Heijde score.

### Greater propensity of RA SF CD19^+^CD24^hi^CD27^+^ B cells to produce RANKL

**Figure 4:**
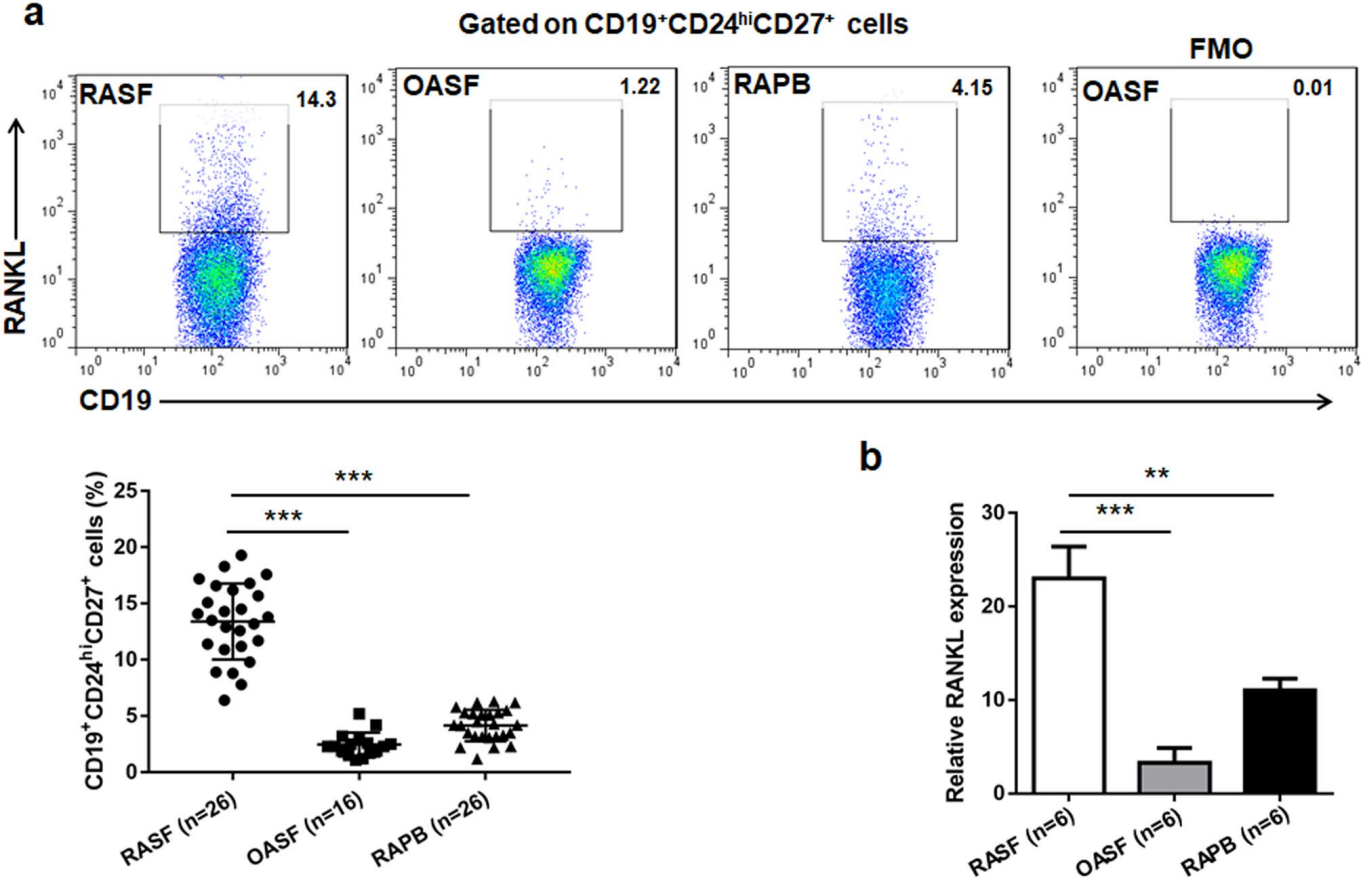
Expression of RANKL by CD19^+^CD24^hi^CD27^+^ B cells was increased in SF from RA patients. Expressions of RANKL in CD19^+^CD24^hi^CD27^+^ B cells from RA SF, OA SF and RA PB were assessed by surface staining and flow cytometry (**a**). Fluorescence-minus-one (FMO) controls were used to determine positive/negative boundaries (**a**). RANKL mRNA expression was assessed by real-time PCR in flow cytometry-sorted CD19^+^CD24^hi^CD27^+^ B cells between six OA patients and six RA patients (**b**). GAPDH serves as an internal standard.

Our previous study has found that RANKL expression of CD19^+^CD24^hi^CD27^+^ B cells from PB of patients with RA was higher as compared to healthy donors^18^. We next sought to determine whether CD19^+^CD24^hi^CD27^+^ B cells in SF from RA patients could produce more RNAKL than that in PB. Indeed, we observed that, compared with CD19^+^CD24^hi^CD27^+^ B cells from OA SF and RA PB, markedly higher percentages of CD19^+^CD24^hi^CD27^+^RANKL^+^ B cells in the SF of patients with RA (Fig. 4a). Furthermore, we also evaluated the levels of RANKL transcripts in CD19^+^CD24^hi^CD27^+^ B cells in RA SF and PB and OA SF by real-time quantitative PCR. As would be expected, in RA SF, the RANKL expression level was increased in CD19^+^CD24^hi^CD27^+^ B cells compared with RA PB and OA SF (Fig. 4b). Altogether, these results clearly demonstrated that the proportion of RANKL-producing CD19^+^CD24^hi^CD27^+^ B cells increased in RA SF.

### SF CD19^+^CD24^hi^CD27^+^ B cells promoted osteoclast differentiation in RA patients

**Figure 5:**
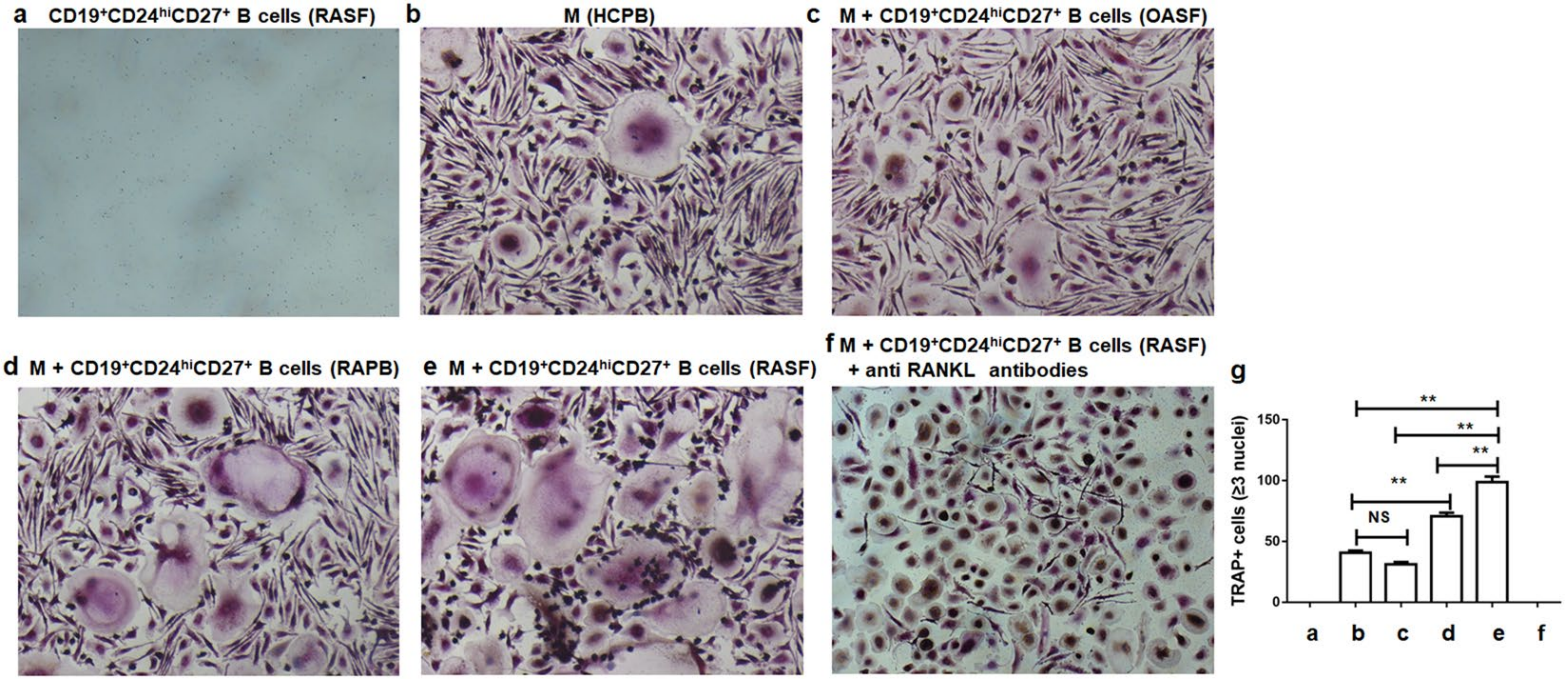
CD19^+^CD24^hi^CD27^+^ B cells in RA patients promoted osteoclast differentiation. Sorted RA SF CD19^+^CD24^hi^CD27^+^ B cells were cultured alone (**a**). Freshly isolated HC PBMCs (5 × 10^5^ per well in 1 ml on a 12-well plate) were cultured in complete αMEM to allow adhering for 2 h in the incubator. Then non-adherent cells were removed to get the adherent osteoclast precursors (> 90% CD14^+^). After that, the adherent cells were cultured alone (**b**) or co-cultured with 1 × 10^5^ OA SF CD19^+^CD24^hi^CD27^+^ B cells (**c**) or with RA PB 1 × 10^5^ CD19^+^CD24^hi^CD27^+^ B cells (**d**) or with RA SF 1 × 10^5^ CD19^+^CD24^hi^CD27^+^ B cells (**e**) in the presence of 30 ng rhM-CSF and 25 ng rhRANKL for 21 days or the addition of 100 ng anti RANKL antibodies for RANKL neutralization experiments (**f**) and stained for tartrate-resistant acid phosphatase (TRAP). TRAP positive multinucleate cells (more than three nuclei) were counted. Original magnification × 200. (**g**) Analysis of the number of TRAP+ multinucleate cells. All data were representative of three independent experiments. Statistical analyses between groups were performed using ANOVA. **p < 0.01; NS, not significant.

PB monocytes were used as osteoclast precursors (OCPs) and could differentiate into osteoclasts (OCs) in the presence of RANKL and M-CSF^25^. To further assess the effect of SF CD19^+^CD24^hi^CD27^+^ B cells on the induction of osteoclastogenesis, we first performed in vitro coculture experiments using CD19^+^CD24^hi^CD27^+^ B cells and CD14^+^ monocytes coculture. CD19^+^CD24^hi^CD27^+^ B cells from SF of RA patients cocultured with monocytes isolated from a healthy donor, and these cells were compared to RA PB CD19^+^CD24^hi^CD27^+^ B cells and OA SF CD19^+^CD24^hi^CD27^+^ B cells. In the cocultures, CD19^+^CD24^hi^CD27^+^ B cells and CD14^+^ monocytes were mixed in the presence of M-CSF and RANKL with relatively low concentration. After 21 days of culture, the cells were evaluated by TRAP activity assay. Indeed, TRAP+ multinucleated OCs were significantly increased in co-culture monocytes with RA SF CD19^+^CD24^hi^CD27^+^ B cells compared with RA PB CD19^+^CD24^hi^CD27^+^ B cells, co-culture with OA SF CD19^+^CD24^hi^CD27^+^ B cells and monocytes alone (Fig. 5b–e, g). As compared to OA SF, we found co-culture with CD19^+^CD24^hi^CD27^+^ B cells from RA PB to have increased numbers of OCs (Fig. 5d, e, g). No TRAP-positive cells were observed in the CD19^+^CD24^hi^CD27^+^ B cells alone cultures (Fig. 5a). We further investigated whether CD19^+^CD24^hi^CD27^+^ B cells from synovial fluid of RA patients promoted osteoclast formation in a RANKL-dependent manner. We added anti RANKL antibodies to the aforementioned coculture system of RA SF CD19^+^CD24^hi^CD27^+^ B cells and CD14+ monocytes. The results showed that RA SF CD24^hi^CD27^+^ B cells can’t induce osteoclast formation by the addition of anti RNAKL antibodies (Fig. 5f). In summary, these results demonstrated that CD19^+^CD24^hi^CD27^+^ B cells in SF from RA patients were a much more significant source of RANKL than that in RA PB and had a greater propensity to support OC differentiation in a RANKL-dependent manner.

## Discussion

In the present study, we have shown that CD19^+^CD24^hi^CD27^+^ B cell numbers were higher in RA SF than that in OA SF and RA PB. Consistently, the percentage of RA SF CD19^+^CD24^hi^CD27^+^ B cells was positively correlated with swollen joint counts, tender joint counts, DAS28 and SHS. Moreover, the proportions of SF CD19^+^CD24^hi^CD27^+^ B cells were increased in ACPA+ RA patients. Our data demonstrate that CXCL13, CXCL12 and CCL19 may promote migration of CD19^+^CD24^hi^CD27^+^ B cells from PB to SF in patients with RA. We further showed that level of RANKL expression in RA SF CD19^+^CD24^hi^CD27^+^ B cells was markedly elevated compared to those of CD19^+^CD24^hi^CD27^+^ B cells from OA SF and RA PB. Finally, we demonstrated that CD19^+^CD24^hi^CD27^+^ B cells from RA SF could promote osteoclast differentiation in RA patients. Taken together, our results suggest that accumulation of SF CD19^+^CD24^hi^CD27^+^ B cells was associated with bone destruction in RA patients.

RA is characterized by chronic inflammation of multiple joints and subsequent joint destruction, which directly lead to joint deformities^26^. The mechanisms of immune-mediated bone destruction in RA are complex^2^. Bone destruction in RA patients has been linked to osteoclast formation for more than 35 years^27^. Earlier studies suggested that synovial fibroblasts and activated T cells were the major source of RANKL^6,28^, which has an essential role in supporting osteoclastogenesis^4^. However, recent evidence indicated that B cells were also capable of producing RANKL and involved in bone destruction^9^. It has been reported that B cell-targeted therapy-rituximab can inhibit the progression of structural joint damage in RA patients^29^. Nevertheless, the specific mechanism of the involvement of B cells in bone erosion has not been well studied. This study highlighted RA SF CD19^+^CD24^hi^CD27^+^ B cells produced RANKL in quantities exceeding that produced by RA PB and OA SF CD19^+^CD24^hi^CD27^+^ B cells, and RA SF CD19^+^CD24^hi^CD27^+^ B cells were found to promote OC differentiation in a RANKL-dependent manner.

Autoimmune diseases often result from an imbalance between regulatory and pathogenic immune cells. Takayanagi et al.^30^ reported that pathogenic conversion of Foxp3 + T cells into TH17 cells has a key role in the pathogenesis of autoimmune arthritis. Our previous study has demonstrated PB regulatory B10 cells converted into osteoclast-priming cells in patients with RA^18^. Human PB CD19^+^CD24^hi^CD27^+^ B cells were proved to secrete IL-10, which was defined as B10 cells^14^. Recent several studies showed that CD19^+^CD24^hi^CD27^+^ B cells displayed a decrease in numbers and impaired immunosuppressive capacity in RA and some other autoimmune disease patients^15,17^. In this study, we shown that migration of CD19^+^CD24^hi^CD27^+^ B cells from PB to the SF in patients with RA explained, at least in part, the decrease in the number of PB CD19^+^CD24^hi^CD27^+^ B cells in patients with RA. Meanwhile, the high expression of RANKL in RA SF CD19^+^CD24^hi^CD27^+^ B cells might lead to impaired immunosuppressive functions of CD19^+^CD24^hi^CD27^+^ B cells.

The ongoing recruitment of inflammatory cells into the RA synovial microenvironment sustains a persistent inflammatory lesion. Numerous studies have demonstrated that chemokines potently promote the migration and activation of pathogenic cells^24,31^. Recent study has shown that CXCL13 and CCL20 might play major roles in the recruitment of B cells within the inflamed synovium^24^. We found that B cell-related chemokines, CXCL10, CXCL12, CXCL13, CCL19, CCL20 and CCL21 expression increased in RF from RA patients. Further, we confirmed that CXCL12, CXCL13, and CCL19 may play important roles in the migration of PB CD19^+^CD24^hi^CD27^+^ B cells to SF of RA patients. The limitation of this study was that these chemokine receptors on CD19^+^CD24^hi^CD27^+^ B cells were not measured, which influenced the assay of cell migration. However, several previous studies have shown that CXCL12, CXCL13, and CCL19 corresponding receptors, CXCR4, CXCR5 and CCR7 have high levels in B cells from RA SF^22,32,33^. Previous studies have reported CCR7 and its ligands play an important role in angiogenesis in RA synovial tissue^34^. Rao et al.^35^ have demonstrated that a Peripheral T helper (TPH) cell subset promotes B-cell responses in RA, which was associated with the expression of CXCL13 and CXCR5. RA SF B cells expressed lower amounts of CCR6 and CXCR5, which increased B-cell migration from PB^24^. Interestingly, recent studies suggested some chemokines promoted RANKL expression and have potential roles in osteoclastogenesis of RA^36,37^. Then, whether these chemokines are involved in bone destruction still needs to be defined.

Many different cell types can produce RANKL, and each of them could potentially contribute to bone destruction in RA patients. Mounting evidence indicates that there are tight relationships between B lymphocytes and bone turnover under pathological conditions^38^. Yeo et al.^9^, reported, for the first time, that B cells infiltrating the synovial fluid of RA patients were the major source of RANKL. Subsequently, they identified a population of FcRL4^+^ B cells that produced RANKL participating in bone erosion in RA patients^10^. Others have shown that RANKL expression was mainly restricted to memory B cells (CD19^+^CD27^+^)^11,12^. In the present work, we observed that CD19^+^CD24^hi^CD27^+^ B cells isolated from SF of patients with RA could promote osteoclast differentiation by up-regulated expression of RANKL. Meanwhile, CD19^+^CD24^hi^CD27^+^ B cells are a major subset of memory B cells. Our data showed that the proportion of SF CD19^+^CD24^hi^CD27^+^ B cells was positively correlated with the hand radiograph progression score SHS. Similar results were found that bone resorption was associated with the frequency of CD5 + B cells in RA patients^39^. ACPA is closely related to osteoclast formation and RA bone erosion^40,41^. We observed significantly higher frequencies of SF CD19^+^CD24^hi^CD27^+^ B cells in ACPA+ RA patients compared to ACPA− patients. Previous study has also demonstrated that levels of synovial CD19^+^ B cells were markedly higher in ACPA+ patients than ACPA− RA patients, and this was associated with elevated serum CXCL13 levels^42^.

At present, the phenotype of human regulatory B10 cells is still controversial. The reported human B10 cells phenotypes are CD19^+^CD24^hi^CD27^+^ B cells or CD19^+^CD24^hi^CD38^hi^ B subset^14,43^. Our previous study found that under the condition of rheumatoid inflammation, PB CD19^+^CD24^hi^CD27^+^ B cells could convert into osteoclast-priming cells^18^. This study confirmed that CD19^+^CD24^hi^CD27^+^ B cells in SF expressed RANKL higher than that in PB and could promote osteoclast differentiation. Therefore, whether CD19^+^CD24^hi^CD27^+^ B cells can represent IL-10-secreting B cells and their role in the pathogenesis and treatment of RA needs to be further confirmed.

Our present study has several limitations. First, we did not compare the migratory capacity of RA SF and PB CD19^+^CD24^hi^CD27^+^B cells. We schedule comprehensively studying the migratory capacity of these cells in vitro and in vivo in the near future. Second, the relationship between CD19^+^CD24^hi^CD27^+^B cells and other B cell subsets in the RA serum/SF was not assessed. These relationships may give more insight to the potential contributions to joint destruction.

In conclusion, our study shed light on a pathogenic role of accumulated SF CD19^+^CD24^hi^CD27^+^ B cells in bone destruction associated with RA patients through production of RANKL. Maintaining beneficially regulatory functions of CD19^+^CD24^hi^CD27^+^ B cells and inhibiting their pathogenic conversion would be a major advance over present therapies for this devastating disease.

## Materials and methods

All methods were performed in accordance with the relevant guidelines and regulations (i.e. Declaration of Helsinki).

### Patients and controls tissue specimens

SF and paired PB samples were obtained from 26 RA patients and 16 osteoarthritis (OA) patients, and healthy control (HC) PB samples were obtained from 20 sex- and age-matched healthy donors. In addition, we collected synovial fluid from 10 ACPA− RA patients. SF was aspirated from swollen joints under palpation or ultrasound guidance. All these RA patients fulfilled the 1987 revised classification criteria of American College of Rheumatology (ACR)^44^. The study protocols were approved by the Institutional Medical Ethics Review Board of the People's Hospital of China Three Gorges University, and all participants gave informed, written consent.

### Flow cytometry and cell sorting

PB mononuclear cells (PBMCs) and SF mononuclear cells (SFMCs) were isolated by density gradient centrifugation. To detect the proportion of CD19^+^CD24^hi^CD27^+^ B cells and the expression of RANKL in CD19^+^CD24^hi^CD27^+^ B cells of PBMCs or SFMCs, the cells were isolated by density gradient centrifugation and then were stained with mouse monoclonal antibodies as follow: CD19-APC/Cy7 (BioLegend, San Diego, CA, USA), CD24-FITC (eBioscience, San Diego, CA, USA), CD27-APC (eBioscience), RANKL-PE (BioLegend). FMO controls were included. Data was acquired on a FACS Arial II flow cytometer (Becton Dickinson, NJ, USA) and analysed using FlowJo software.

To isolate CD19^+^CD24^hi^CD27^+^ B cells, PBMCs were stained with mouse anti-CD19-APC/Cy7, CD24-PE (eBioscience), CD27-APC, then the aimed cell populations were sorted by flow cytometry. Sorted CD19^+^CD24^hi^CD27^+^ B cells had a purity of > 95%. These sorted cells were subsequently subjected to reverse transcription-polymerase chain reaction (RT-PCR) or osteoclast differentiation assay.

### Enzyme-linked immunosorbent assay (ELISA)

Human CXCL10, CXCL12, CXCL13, CCL19, CCL20 and CCL21 were measured by ELISA, according to the manufacturer’s instructions (R&D Systems). The serum and synovial fluids were from RA (n = 26) and OA (n = 16) patients.

### Measurement of bone destruction

A total of 26 sets of hand radiographs from RA patients were obtained within 1 weeks of synovial fluid and peripheral blood drawing. Radiographs of the hands were scored, using the modified Sharp/van der Heijde score (SHS)^45^, by an experienced professional reader (Shulin Song) who was blinded with regard to the clinical and experimental data.

### RT-PCR and realtime PCR

RNA was extracted from sorted B cell subsets using RNeasy mini kit (Qiagen). RT-PCR was performed as previously described^18^. The oligo(dT)-primed cDNA was synthesized using the RevertAid FirstStrand kit (Fermentas, Glen Burnie, MD, USA) according to the manufacturer’s directions. The resultant cDNA was subjected to real time PCR analyses.

Real time PCR was performed on the 7900HT Real-Time PCR System (Applied Biosystems) using SYBR Green Master Mix (Applied Biosystems, Foster City, CA, USA). The following primers were used for real time PCR of RANKL splice variants: forward 5′-TCGTTGGATCACAGCACATCA-3′ and reverse 5′-TATGGGAACCAGATGGGATGTC-3′. Amplification of the housekeeping gene GAPDH was used as an internal control. Gene expression was quantified relative to the expression of GAPDH, and normalized to control by standard 2^−△△CT^ calculation.

### Osteoclast differentiation assay

As described above, PBMCs and sorted SF CD19^+^CD24^hi^CD27^+^ B cells or PB CD19^+^CD24^hi^CD27^+^ B cell subsets were prepared. Freshly isolated PBMCs (5 × 10^5^ per well in 1 ml on a 12-well plate) were cultured in complete α-minimal essential medium (10% FBS, 1% penicillin–streptomycin) to allow adhering for 2 h in the incubator. Then non-adherent cells were removed to get the adherent osteoclast precursors (> 90% CD14^+^). After that, the adherent cells were cultured alone or co-cultured with 1 × 10^5^ SF CD19^+^CD24^hi^CD27^+^ B cells (5:1) or with PB 1 × 10^5^ CD19^+^CD24^hi^CD27^+^ B cells (5:1) in the presence of 30 ng recombinant human macrophage colony-stimulating factor (rhM-CSF) (Peprotech) and 25 ng rhRANKL (R&D systems). For RANKL neutralization experiments, 100 ng neutralizing antibody against human RANKL (Sino Biologicals) was added during the aforementioned co-culture system. Cultures were fed every 3 days with fresh medium. On day 21, cells were stained for tartrate-resistant acid phosphatase (TRAP) using the Leukocyte Acid Phosphatase kit according to the instructions of the manufacturer (Sigma). TRAP positive multinucleate cells (three or more nuclei) were counted under a microscope.

### Statistics

Means are shown on dot plots. The distribution of continuous variables was tested with the Shapiro–Wilk statistic. Means between two groups were assessed by a two-tailed unpaired Student *t* test when the data distribution was normal and the nonparametric Mann–Whitney rank sum test when it was skewed. One-way ANOVA with post hoc Dunnett multiple-comparisons test was performed to evaluate three data sets. Spearman’s correlation coefficient was used to study the correlation between different continuous parameters. *p* values less than 0.05 were considered statistically significant. All statistical analyses were performed using IBM SPSS Statistics version 20.0 (IBM, Armonk, NY, USA).

## References

[1] McInnes IB, Schett MD (2011). The pathogenesis of rheumatoid arthritis. N. Engl. J. Med..

[2] Schett G, Gravallese E (2012). Bone erosion in rheumatoid arthritis: mechanisms, diagnosis and treatment. Nat. Rev. Rheumatol..

[3] Hirayama T, Danks L, Sabokbar A, Athanasou NA (2002). Osteoclast formation and activity in the pathogenesis of osteoporosis in rheumatoid arthritis. Rheumatology.

[4] Kobayashi K (2000). Tumor necrosis factor a stimulates osteoclast differentiation by a mechanism independent of the ODF/RANKL-RANK interaction. J. Exp. Med..

[5] Lacey DL (1998). Osteoprotegerin ligand is a cytokine that regulates osteoclast differentiation and activation. Cell.

[6] Takayanagi H (2000). Involvement of receptor activator of nuclear factor kappaB ligand osteoclast differentiation factor in osteoclastogenesis from synoviocytes in rheumatoid arthritis. Arthritis Rheum..

[7] Gravallese EM (2000). Synovial tissue in rheumatoid arthritis is a source of osteoclast differentiation factor. Arthritis Rheum..

[8] Kotake S (2001). Activated human T cells directly induce osteoclastogenesis from human monocytes. Arthritis Rheum..

[9] Yeo L (2011). Cytokine mRNA profiling identifies B cells as a major source of RANKL in rheumatoid arthritis. Ann. Rheum. Dis..

[10] Yeo L (2015). Expression of FcRL4 defines a pro-inflammatory, RANKL-producing B cell subset in rheumatoid arthritis. Ann. Rheum. Dis..

[11] Meednu N (2016). Production of RANKL by memory B cells: a link between B cells and bone erosion in rheumatoid arthritis. Arthritis Rheumatol..

[12] Ota Y (2016). Generation mechanism of RANKL(+) effector memory B cells: relevance to the pathogenesis of rheumatoid arthritis. Arthritis Res. Ther..

[13] Sun W (2018). B cells inhibit bone formation in rheumatoid arthritis by suppressing osteoblast differentiation. Nat. Commun..

[14] Iwata Y (2011). Characterization of a rare IL-10-competent B-cell subset in humans that parallels mouse regulatory B10 cells. Blood.

[15] Daien CI (2014). Regulatory B10 cells are decreased in patients with rheumatoid arthritis and are inversely correlated with disease activity. Arthritis Rheumatol..

[16] Bingbing Z (2012). Decrease in proportion of CD19^+^CD24^hi^CD27^+^ B cells and impairment of their suppressive function in Graves’ disease. PLoS ONE.

[17] Jin L, Chen W, Yue L (2013). Peripheral CD24^(hi)^CD27^(+)^CD19^(+)^B cells subset as a potential biomarker in naive systemic lupus erythematosus. Int. J. Rheum. Dis..

[18] Hu F (2017). Pathogenic conversion of regulatory B10 cells into osteoclast-priming cells in rheumatoid arthritis. J. Autoimmun..

[19] Mellado M (2015). T cell migration in rheumatoid arthritis. Front. Immunol..

[20] Iwamoto T, Okamoto H, Toyama Y, Momohara S (2008). Molecular aspects of rheumatoid arthritis: chemokines in the joints of patients. FEBS J..

[21] Pandya JM (2017). Blood chemokine profile in untreated early rheumatoid arthritis: CXCL10 as a disease activity marker. Arthritis Res. Ther..

[22] Henneken M, Dorner T, Burmester GR, Berek C (2005). Differential expression of chemokine receptors on peripheral blood B cells from patients with rheumatoid arthritis and systemic lupus erythematosus. Arthritis Res. Ther..

[23] Sellam J (2013). CCL19, a B cell chemokine, is related to the decrease of blood memory B cells and predicts the clinical response to rituximab in patients with rheumatoid arthritis. Arthritis Rheum..

[24] Armas-Gonzalez E (2018). Role of CXCL13 and CCL20 in the recruitment of B cells to inflammatory foci in chronic arthritis. Arthritis Res. Ther..

[25] Akagawa KS (1996). Generation of CD1^+^RelB^+^ dendritic cells and tartrate-resistant acid phosphatase-positive osteoclast-like multinucleated giant cells from human monocytes. Blood.

[26] Ostrowska M, Maśliński W, Prochorec-Sobieszek M, Nieciecki M, Sudoł-Szopińska I (2018). Cartilage and bone damage in rheumatoid arthritis. Reumatol./Rheumatol..

[27] Bromley M, Woolley DE (1984). Chondroclasts and osteoclasts at subchondral sites of cartilage erosion in the rheumatoid joint. Arthritis Rheum..

[28] Sato K (2006). Th17 functions as an osteoclastogenic helper T cell subset that links T cell activation and bone destruction. J. Exp. Med..

[29] Keystone E (2009). Rituximab inhibits structural joint damage in patients with rheumatoid arthritis with an inadequate response to tumour necrosis factor inhibitor therapies. Ann. Rheum. Dis..

[30] Komatsu N (2013). Pathogenic conversion of Foxp3^+^ T cells into T_H_17 cells in autoimmune arthritis. Nat. Med..

[31] Hirota K (2007). Preferential recruitment of CCR6-expressing Th17 cells to inflamed joints via CCL20 in rheumatoid arthritis and its animal model. J. Exp. Med..

[32] Moschovakis GL (2019). The chemokine receptor CCR7 is a promising target for rheumatoid arthritis therapy. Cell Mol. Immunol..

[33] Nanki T (2009). Chemokine receptor expression and functional effects of chemokines on B cells: implication in the pathogenesis of rheumatoid arthritis. Arthritis Res. Ther..

[34] Pickens SR (2011). Characterization of CCL19 and CCL21 in rheumatoid arthritis. Arthritis Rheum..

[35] Rao DA (2017). Pathologically expanded peripheral T helper cell subset drives B cells in rheumatoid arthritis. Nature.

[36] Lee EY (2011). Potential role and mechanism of IFN-gamma inducible protein-10 on receptor activator of nuclear factor kappa-B ligand (RANKL) expression in rheumatoid arthritis. Arthritis Res. Ther..

[37] Lee J (2017). Stimulation of osteoclast migration and bone resorption by C–C chemokine ligands 19 and 21. Exp. Mol. Med..

[38] Horowitz MC, Fretz JA, Lorenzo JA (2010). How B cells influence bone biology in health and disease. Bone.

[39] Engelmann R, Wang N, Kneitz C, Müller-Hilke B (2015). Bone resorption correlates with the frequency of CD5^+^ B cells in the blood of patients with rheumatoid arthritis. Rheumatology.

[40] Syversen SW (2008). High anti-cyclic citrullinated peptide levels and an algorithm of four variables predict radiographic progression in patients with rheumatoid arthritis: results from a 10-year longitudinal study. Ann. Rheum. Dis..

[41] Harre U (2012). Induction of osteoclastogenesis and bone loss by human autoantibodies against citrullinated vimentin. J. Clin. Invest..

[42] Orr C (2017). Synovial immunophenotype and anti-citrullinated peptide antibodies in rheumatoid arthritis patients: relationship to treatment response and radiologic prognosis. Arthritis Rheumatol..

[43] Blair PA (2010). CD19^+^CD24^hi^CD38^hi^ B cells exhibit regulatory capacity in healthy individuals but are functionally impaired in systemic lupus erythematosus patients. Immunity.

[44] Arnett FC (1988). The American Rheumatism Association 1987 revised criteria for the classification of rheumatoid arthritis. Arthritis Rheum..

[45] Van der Heijde DM (2000). How to read radiographs according to the Sharp/van der Heijde method. J. Rheumatol..

